# Evaluation of Students' Self‐Perceived Competence in Dental Trauma Management in a Case‐Based Simulation Training

**DOI:** 10.1111/edt.70021

**Published:** 2025-09-30

**Authors:** Marcel Reymus, Christina Fotiadou, Falk Schwendicke, Ralf Krug, Christian Diegritz

**Affiliations:** ^1^ Department of Conservative Dentistry, Periodontology and Digital Dentistry LMU University Hospital, LMU Munich Munich Germany; ^2^ Department of Conservative Dentistry and Periodontology University Hospital Würzburg Würzburg Germany

**Keywords:** 3D printing, case‐based learning (CBL), dental traumatology, endodontic education, self‐perceived competence, simulation

## Abstract

**Objectives:**

The purpose of this study was to evaluate the impact of a case‐based hands‐on training session on dental students' self‐perceived competence in managing traumatic dental injuries (TDI) using an innovative 3D‐printed model.

**Methods:**

Fifth‐year undergraduate students were invited to voluntarily participate in a hands‐on training on dental traumatology. Participants worked through a case‐based multi‐injury trauma using a specially designed 3D‐printed model, allowing them to practice examination, diagnosis, and treatment procedures in a realistic clinical scenario. A questionnaire evaluating self‐perceived competence in various TDI management domains was conducted immediately before, directly after, and 12 months after the training. Pre‐ and post‐training scores were analyzed statistically.

**Results:**

A total of 42 students participated in the training over two consecutive years. Thirty students participated in the evaluation 12 months after the training. Participants demonstrated a statistically significant increase in self‐perceived competence for most assessed areas directly after the training, which kept true even after 12 months. The only exception was “worries about complications during clinical management,” which showed no significant change.

**Conclusion:**

Simulation‐based hands‐on training substantially enhanced dental students' self‐perceived competence in managing TDI.

## 1 Introduction

The clinical management of traumatic dental injuries (TDI) falls within the scope of endodontics alongside other disciplines such as oral surgery and pediatric dentistry [1]. The Undergraduate Curriculum Guidelines of the European Society of Endodontology acknowledge the importance of TDI management as an essential component of the endodontic curriculum [2]. However, teaching TDI presents unique challenges. Due to the acute nature of TDIs—most commonly affecting children—the direct involvement of undergraduate students in clinical cases is often not feasible. Consequently, education in this field primarily relies on theoretical lectures that focus on classification systems and treatment protocols, lacking the essential hands‐on experience.

Research suggests that students' knowledge of dental traumatology is relatively low, and traditional lecture‐based teaching methods only lead to short‐term improvements in knowledge retention [3]. This lack of knowledge and competence extends to clinical practice, as multiple studies have shown that practicing dentists often have inadequate awareness and proficiency in managing TDI [4, 5, 6]. This is particularly concerning, as timely and appropriate treatment is crucial for achieving optimal patient outcomes [7]. Consequently, many dentists report a lack of self‐perceived competence in managing TDI cases. Self‐perceived competence refers to an individual's belief in their own abilities, whereas self‐efficacy—a psychological construct introduced by Albert Bandura [8]—relates to one's confidence in their capability to perform tasks and achieve desired outcomes in the future. In the context of endodontics, high self‐efficacy equips practitioners with the assurance to perform complex procedures, handle complications, and make informed decisions, ultimately improving patient outcomes [9, 10, 11, 12]. However, one source of self‐efficacy is personal success experiences or vicarious experiences from colleagues who have similar backgrounds or training. As laid out, clinical TDI training of undergraduate students seems unrealistic in many dental schools around Europe [2]. To address these limitations, innovative educational methods are explored to increase students' hands‐on experience and self‐perceived competence and thus self‐efficacy. One such approach is the integration of simulation‐based training using 3D‐printed models. Several studies have highlighted the benefits of such training [13, 14]. At the Department of Conservative Dentistry, Periodontology and Digital Dentistry of the University of Munich, Germany, a 3D‐printed model specifically designed for hands‐on training for TDI has been developed and incorporated into the undergraduate curriculum. Initial evaluations by students indicated that the model provided a realistic representation of clinical scenarios, enabling diagnosis, treatment planning, and clinical management exercises [15]. Additive manufacturing, or 3D printing, enables faculty and their members to create specific training models that may not be commercially available on their own. As a result, students benefit from innovative new training interventions that can improve their clinical performance. Several such models have recently been described in the literature [16, 17, 18]. The aim of this study was to evaluate the effect of simulation‐based hands‐on training on the self‐perceived competence of undergraduate dental students using a 3D‐printed model.

## 2 Materials and Methods

This study was approved by the Ethics Committee of the Medical Faculty of the University of Munich, Germany, (24‐0567‐KB). Informed consent was obtained from all participants. Fifth‐year undergraduate dental students were invited to participate in an elective hands‐on dental trauma training course. All participants had previously attended lectures on dental traumatology as part of the undergraduate curriculum at the Department of Conservative Dentistry, Periodontology and Digital Dentistry of the University of Munich. The theoretical component included 6 h of lectures covering the classification and clinical management of TDI, encompassing diagnosis, management of complications, and follow‐up protocols. For the hands‐on training component, students received a brief introduction outlining the course structure, without additional theoretical input on dental traumatology. The students were divided into groups of three to four and completed the course independently. They were allowed to request assistance from one of the three supervising dentists present whenever they had practical or theoretical questions.

Each group was provided with a case‐based manuscript simulating a real‐life scenario. The simulation began with a virtual phone call from a mother reporting that her 16‐year‐old son had been involved in an accident, was bleeding from the mouth, and had one tooth completely avulsed. The participants were required to provide the mother with appropriate first aid instructions. Subsequently, they proceeded to the next phase, where they virtually “received” the patient in their dental office. At this stage, the students were presented with a 3D‐printed model of the patient's dentition, along with additional clinical information, including pulp sensitivity tests, percussion responses, and bleeding sites. Furthermore, two‐dimensional radiographic images of the model were displayed on computer screens, covering the region from the left to the right maxillary canine. The manuscript given to the participants is presented in Appendix 1. Participants were tasked with diagnosing each affected tooth, planning and performing appropriate treatment, and establishing a suitable follow‐up regimen.

**FIGURE 1:**
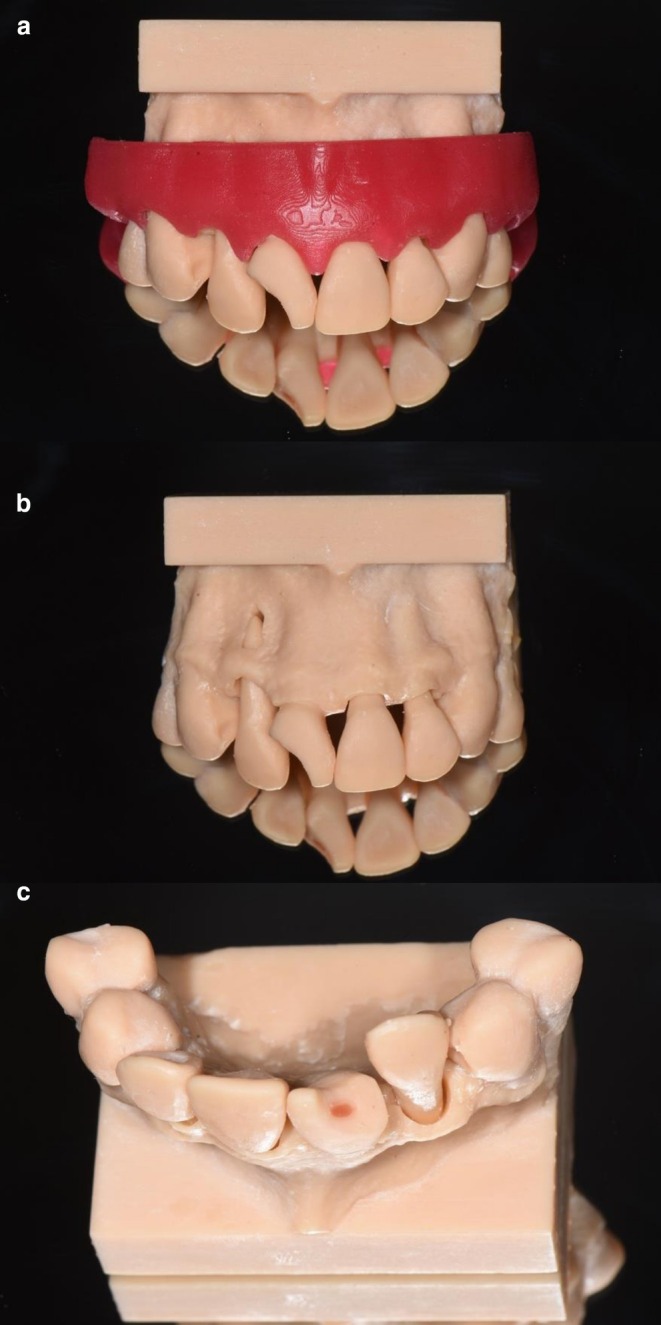
(a–c) Photographs of the 3D‐printed model, with and without gingival mask, showing the dislocated tooth 12 and the crown fracture with pulp exposure on tooth 11.

The 3D‐printed model used in the training was specifically designed for the department's educational purposes and is depicted in Figure 1a–c. The model was fully designed and manufactured within the department, using a CBCT scan of a maxilla from an actual patient with no TDI or other anomalies. DICOM data were imported into the software 3D Slicer (slicer.org) and converted into STL files. These STL files were then processed in Blender (blender.org) to simulate multiple TDIs. Tooth 12 was designed to mimic a lateral luxation, with the apex perforating the buccal cortical bone; it was also made removable. Tooth 11 represented a crown fracture with pulp exposure, for which red dental wax was used to simulate a pulp that could be partially removed using a dental bur to train a partial pulpotomy. Tooth 21 was fabricated as a separate, removable component to simulate an avulsed tooth. Tooth 22 represented an intra‐alveolar root fracture, with the coronal fragment also rendered separate and removable. The final model was additively manufactured (FabPro 1000, 3D Systems) using a radiopaque dental resin (Figure 1b,c) (NextDent, Soesterberg, the Netherlands). The model was processed according to the manufacturer's instructions, including cleaning in an ultrasonic bath of isopropanol and subsequent post‐curing in the LC‐3D Print Box (NextDent).

The self‐perceived competences were assessed by a self‐composed questionnaire with a four‐point Likert scale adapted for TDI based on the publication by Baaij and Özok [9]. The case‐based learning format (CBL) as well as the questionnaire were evaluated during the Academic Teacher Training Course of the medical faculty of the University of Munich, Germany. For this purpose, the original questionnaire by Baaij et al. was adapted, which means the questions concerning root canal treatment were changed to treatment of TDI. This adapted questionnaire was thoroughly discussed with other teachers from the medical faculty, all of whom have experience in designing such survey‐based studies. However, care was taken to ensure that it was not changed too much from the original questionnaire to allow comparison between the self‐perceived competence in root canal treatment and TDI or other future studies regarding this question.

Self‐perceived competence was assessed across the following domains: providing instructions to a layperson on handling a TDI at the scene of an accident (Instruction), conducting a medical examination of a traumatized patient (Examination), establishing an accurate diagnosis (Diagnosis), performing clinical management of a TDI (Therapy), evaluating the prognosis of a TDI (Prognosis), and developing an appropriate recall regimen (Recall). Additionally, participants were asked to evaluate their perceived competence compared to that of a practicing dentist (Competence), their worries about facing complications during treatment (Worries), and their confidence in managing such a complication (Complication). The questionnaire used for data collection is presented in Table 1. The questionnaire was handed out to the participants before and directly after the training, as well as 12 months after the training. Statistical analyses were performed using SPSS 30.0 (IBM, Armonk, NY, USA). The chi‐square test was applied to evaluate overall response distributions before and after training within each domain. To assess specific differences between individual response categories within a domain, Fisher's exact test was used. The level of statistical significance was set at *p* < 0.05. No adjustment for multiple testing was performed given the explorative nature of the study.

**TABLE 1:** Questionnaire and results before and after training.

		Not true at all	Hardly true	Moderately true	Exactly true
I feel competent to independently give a patient *instructions* over the phone on what to do after a dental accident	X	2 (X − Y: 0.494, X − Z: 0.507)	21*′ (X − Y: *p* < 0.05, X − Z: *p* < 0.05)	19 (X − Y: 0.372, X − Z: 0.241)	0*′ (X − Y: *p* < 0.05, X − Z: *p* < 0.05)
	Y	0 (X − Y: 0.494, Y − Z: 1)	0* (X − Y: *p* < 0.05, Y − Z: 1)	14** (X − Y: 0.372, Y − Z: *p* < 0.05)	28*^,^** (X − Y: *p* < 0.05, Y − Z: *p* < 0.05)
	Z	0 (Y − Z: 1, X − Z: 0.507)	0′ (Y − Z: 1, X − Z: *p* < 0.05)	18** (Y − Z: *p* < 0.05, X − Z: 0.241)	12**′ (Y − Z: *p* < 0.05, X − Z: *p* < 0.05)
I feel competent to independently prepare a complete *examination* after a dental injury caused by an accident	X	20*′ (X − Y: *p* < 0.05, X − Z: *p* < 0.05)	21′ (X − Y: 0.379, X − Z: *p* < 0.05)	0*′ (X − Y: *p* < 0.05, X − Z: *p* < 0.05)	20*′ (X − Y: *p* < 0.05, X − Z: *p* < 0.05)
	Y	0*^,^** (X − Y: *p* < 0.05, Y − Z: *p* < 0.05)	26** (X − Y: 0.379, Y − Z: *p* < 0.05)	16* (X − Y: *p* < 0.05, Y − Z: 0.0941)	0*^,^** (X − Y: *p* < 0.05, Y − Z: *p* < 0.05)
	Z	6**′ (Y − Z: *p* < 0.05, X − Z: *p* < 0.05)	6**′ (Y − Z: *p* < 0.05, X − Z: *p* < 0.05)	18′ (Y − Z: 0.0941, X − Z: *p* < 0.05)	6**′ (Y − Z: *p* < 0.05, X − Z: *p* < 0.05)
I feel competent to independently make a correct *diagnosis* of a dental injury caused by an accident	X	30*′ (X − Y: *p* < 0.05, X − Z: *p* < 0.05)	10*′ (X − Y: *p* < 0.05, X − Z: *p* < 0.05)	1*′ (X − Y: *p* < 0.05, X − Z: *p* < 0.05)	30*′ (X − Y: *p* < 0.05, X − Z: *p* < 0.05)
	Y	4* (X − Y: *p* < 0.05, Y − Z: 0.302)	27* (X − Y: *p* < 0.05, Y − Z: 0.807)	11* (X − Y: *p* < 0.05, Y − Z: 0.587)	4* (X − Y: *p* < 0.05, Y − Z: 0.302)
	Z	6′ (Y − Z: 0.302, X − Z: *p* < 0.05)	18′ (Y − Z: 0.807, X − Z: *p* < 0.05)	6′ (Y − Z: 0.587, X − Z: *p* < 0.05)	6′ (Y − Z: 0.302, X − Z: *p* < 0.05)
I feel competent to independently *treat* a dental injury caused by an accident	X	9*′ (X − Y: *p* < 0.05, X − Z: *p* < 0.05)	28*′ (X − Y: *p* < 0.05, X − Z: *p* < 0.05)	4*′ (X − Y: *p* < 0.05, X − Z: *p* < 0.05)	1*′ (X − Y: *p* < 0.05, X − Z: *p* < 0.05)
	Y	0* (X − Y: *p* < 0.05, Y − Z: 1)	8* (X − Y: *p* < 0.05, Y − Z: 1)	25* (X − Y: *p* < 0.05, Y − Z: 1)	9* (X − Y: *p* < 0.05, Y − Z: 1)
	Z	0′ (Y − Z: 1, X − Z: *p* < 0.05)	6′ (Y − Z: 1, X − Z: *p* < 0.05)	18′ (Y − Z: 1, X − Z: *p* < 0.05)	6′ (Y − Z: 1, X − Z: *p* < 0.05)
I feel competent to independently make a *prognosis* for an injured tooth	X	6*′ (X − Y: *p* < 0.05, X − Z: *p* < 0.05)	25* (X − Y: *p* < 0.05, X − Z: 0.151)	11*′ (X − Y: *p* < 0.05, X − Z: *p* < 0.05)	0* (X − Y: *p* < 0.05, X − Z: 1)
	Y	0* (X − Y: *p* < 0.05, Y − Z: 1)	7*^,^** (X − Y: *p* < 0.05, Y − Z: *p* < 0.05)	23* (X − Y: *p* < 0.05, Y − Z: 0.81)	12*^,^** (X − Y: *p* < 0.05, Y − Z: *p* < 0.05)
	Z	0′ (Y − Z: 1, X − Z: *p* < 0.05)	12** (Y − Z: *p* < 0.05, X − Z: 0.151)	18′ (Y − Z: 0.81, X − Z: *p* < 0.05)	0** (Y − Z: *p* < 0.05, X − Z: 1)
I feel competent to independently establish a *recall regime* for an injured tooth	X	7*′ (X − Y: *p* < 0.05, X − Z: *p* < 0.05)	28*′ (X − Y: *p* < 0.05, X − Z: *p* < 0.05)	7*′ (X − Y: *p* < 0.05, X − Z: *p* < 0.05)	0* (X − Y: *p* < 0.05, X − Z: 1)
	Y	0* (X − Y: *p* < 0.05, Y − Z: 0.994)	5* (X − Y: *p* < 0.05, Y − Z: 0.347)	29* (X − Y: *p* < 0.05, Y − Z: 0.299)	8*^,^** (X − Y: *p* < 0.05, Y − Z: *p* < 0.05)
	Z	0′ (Y − Z: 0.994, X − Z: *p* < 0.05)	6′ (Y − Z: 0.347, X − Z: *p* < 0.05)	24′ (Y − Z: 0.299, X − Z: *p* < 0.05)	0** (Y − Z: *p* < 0.05, X − Z: 1)
I consider my *competence* in treating an accidental dental injury to be equal to those of a practicing dentist	X	25*′ (X − Y: *p* < 0.05, X − Z: *p* < 0.05)	15 (X − Y: 0.376, X − Z: 0.807)	2*′ (X − Y: *p* < 0.05, X − Z: *p* < 0.05)	0 (X − Y: 0.116, X − Z: 1)
	Y	4* (X − Y: *p* < 0.05, Y − Z: 0.135)	20 (X − Y: 0.376, Y − Z: 0.632)	14*** (X − Y: *p* < 0.05, Y − Z: *p* < 0.05)	4 (X − Y: 0.116, Y − Z: 0.135)
	Z	0′ (Y − Z: 0.135, X − Z: *p* < 0.05)	12 (Y − Z: 0.632, X − Z: 0.807)	18**′ (Y − Z: *p* < 0.05, X − Z: *p* < 0.05)	0 (Y − Z: 0.135, X − Z: 1)
I am *worried* about encountering complications during the treatment of an accidental dental injury	X	2 (X − Y: 1, X − Z: 0.539)	7′ (X − Y: 0.588, X − Z: *p* < 0.05)	25 (X − Y: 1, X − Z: 1)	8′ (X − Y: 0.194, X − Z: *p* < 0.05)
	Y	3 (X − Y: 1, Y − Z: 0.261)	10 (X − Y: 0.588, Y − Z: 0.195)	26 (X − Y: 1, Y − Z: 1)	3 (X − Y: 0.194, Y − Z: 0.261)
	Z	0 (Y − Z: 0.261, X − Z: 0.539)	12′ (Y − Z: 0.195, X − Z: *p* < 0.05)	18 (Y − Z: 1, X − Z: 1)	0′ (Y − Z: 0.261, X − Z: *p* < 0.05)
I know how to independently *manage complications* during the treatment of an accidental dental injury	X	8*′ (X − Y: *p* < 0.05, X − Z: *p* < 0.05)	32*′ (X − Y: *p* < 0.05, X − Z: *p* < 0.05)	2*′ (X − Y: *p* < 0.05, X − Z: *p* < 0.05)	0 (X − Y: 0.0551, X − Z: 1)
	Y	1* (X − Y: *p* < 0.05, Y − Z: 1)	13* (X − Y: *p* < 0.05, Y − Z: 0.461)	23* (X − Y: *p* < 0.05, Y − Z: 0.81)	5 (X − Y: 0.0551, Y − Z: 0.071)
	Z	0′ (Y − Z: 1, X − Z: *p* < 0.05)	12′ (Y − Z: 0.461, X − Z: *p* < 0.05)	18′ (Y − Z: 0.81, X − Z: *p* < 0.05)	0 (Y − Z: 0.071, X − Z: 1)

*Note:* * mark significant differences between individual categories before (X) and directly after treatment (Y), ** mark significant differences between individual categories directly after and 12 months after treatment (Z), ′ mark significant differences between individual categories before and 12 months after treatment according to Fisher's exact test.

## 3 Results

A total of 42 students (of initially 65 invited students) participated in the hands‐on training and completed the initial questionnaires; of these, 30 also completed the 12‐month follow‐up. The analysis of the three time points (pre‐training, post‐training, and 12‐month follow‐up) indicates a dynamic development in self‐perceived competence.

Initially, a comparison of pre‐ and post‐training data confirmed the significant impact of the intervention. The chi‐square test revealed statistically significant differences (*p* < 0.05) in the overall distribution of responses for the domains “Instruction,” “Examination,” “Report,” “Diagnosis,” “Therapy,” “Prognosis,” “Recall,” “Competence,” and “Complication.” In contrast, no statistically significant difference was found for the domain “Worries” (*p* = 0.388).

Comparing the immediate post‐training data to the 12‐month follow‐up, the chi‐square test showed a statistically significant change in the overall response distribution for most domains (*p* < 0.05), indicating a decrease in self‐perceived competence over time. However, the domains “Complication” (*p* = 0.273) and “Worries” (*p* = 0.813) showed no significant change.

Despite this decline, a lasting benefit was evident. The comparison between pre‐training and 12‐month follow‐up data still yielded statistically significant improvements (*p* < 0.05) for all domains except “Worries.”

A more detailed analysis using Fisher's exact test for individual response categories elaborates on these trends as presented in Table 1. Between the pre‐ and post‐training surveys, significant shifts (*p* < 0.05) were seen in almost all categories, reflecting the immediate positive impact of the training. When analyzing the subsequent decline from post‐training to the 12‐month follow‐up, a significant shift from higher ratings (“exactly true,” “partly true”) to lower ones (“hardly true”) was identified in most domains, such as “Therapy” and “Competence” (*p* < 0.05). However, for the “Complication” domain, no individual category showed a significant change during this period.

Even after 12 months, self‐perceived competence largely remained above pre‐training levels. For instance, the “exactly true” responses for domains like “Instruction” (*p* < 0.05) and “Therapy” (*p* < 0.05) were still significantly higher than before the training. However, for some specific categories, the long‐term benefit was less pronounced. The rating “exactly true” in the “Prognosis” domain at 12 months was no longer significantly different from the pre‐training level indicating that the peak level of confidence had subsided. As with the overall analysis, no significant differences (*p* < 0.539) were observed across any response category within the “Worries” domain throughout the entire study period, except “exactly true” between before training and 12 months after.

## 4 Discussion

Before the training, the students' self‐perception was characterized by low confidence. For nearly all practical domains (e.g., “Instruction,” “Therapy,” “Competence”), the responses were heavily concentrated in the “not true at all” and “hardly true” categories. This indicates a clear, recognized need for training in these areas. The only exception was the “Worries” domain, where students already felt more confident, which is logical as it reflects a personal state rather than a technical skill. The training had a dramatic and immediate positive impact. In the post‐training survey, there was a massive, statistically significant shift across all practical domains. The counts for “not true at all” and “hardly true” dropped to virtually zero. Correspondingly, the responses shifted overwhelmingly to “partly true” and, most notably, “exactly true.” This demonstrates that the training was highly effective in creating an immediate and strong boost in self‐perceived competence. The students left the training feeling significantly more capable and confident. The 12‐month follow‐up is crucial as it reveals the sustainability of this confidence boost. A significant “regression toward the mean” is evident. When comparing the 12‐month data to the post‐training data, there is a clear and statistically significant decline. For most domains, responses moved from “exactly true” back toward “partly true” and “hardly true.” This suggests that the peak confidence achieved immediately after the training is not fully maintained; without reinforcement, a degree of self‐perceived competence is lost over time. Despite the decay, the most important finding is that the 12‐month levels of self‐perceived competence are still significantly higher than the pre‐training levels. For example, in the “Therapy” domain, while the “exactly true” responses dropped from 24 to 6, the number of “not true at all” and “hardly true” responses (2 and 13 respectively) is still vastly lower than the pre‐training numbers (18 and 17). This pattern holds true for all practical skills.

Similar case‐based approaches have been successfully integrated into dental undergraduate curricula [19, 20]. The positive impact of realistic training measures on self‐perceived competence is consistent with previous research [21, 22]. Studies have shown that students around the world often lack confidence in their ability to perform TDI procedures properly [23, 24, 25].

One contributory factor may be limited clinical exposure to TDI. From an educational standpoint, providing real‐time hands‐on learning in TDI emergency situations is challenging. Even experienced clinicians face the stress of treating an anxious child and dealing with equally concerned parents in a high‐pressure context. As a result, involving inexperienced students directly in these emergencies is usually impractical. To address this challenge, models specifically designed for 3D printing can be used by dental clinics. To the best of the authors' knowledge, there are no commercially available models for dental traumatology. Other models described in the literature were considered inappropriate because they are based on animal cadavers [26]. Consequently, a 3D‐printed model derived from a CBCT scan of a real patient was used for hands‐on training in this study [15]. Recent innovations in 3D printing materials have improved the radiopacity and dentin‐like hardness of the model. The model used in this study allowed clinical and radiographic diagnosis and treatment of all simulated TDIs (repositioning, splinting, partial pulpotomy with direct capping). This feature probably made the training more realistic for the students. The addition of a case‐based manuscript involving a 16‐year‐old boy's accident further enhanced the realism and educational impact.

From an instructional perspective, the heightened self‐perceived competence observed among students underscores the value of such a simulation‐based hands‐on training. Such a realistic setting in which students can practice decision‐making, perform procedures, and receive immediate feedback seems to deepen students' understanding of TDI management principles. Such experiential learning is especially valuable in areas like dental traumatology, where it is often difficult to provide sufficient hands‐on experiences during a standard undergraduate curriculum. For this purpose, specially designed 3D‐printed training models seem to be of great advantage. Although only self‐perceived competence was assessed in this study, it is one foundation of self‐efficacy. Furthermore, successful participation in such a training measure with seemingly long‐lasting effects can effectively contribute to students' self‐efficacy. Nevertheless, self‐efficacy should be further investigated in upcoming studies with appropriately designed surveys and study setups.

Several previous studies have described the use of 3D‐printed models for training in dental traumatology. Zafar et al. [27] used a single 3D‐printed tooth simulating an avulsed tooth, which could be used within a typodont model. A recent study described the use of a commercial model with commercially available single teeth for training in TDI [28]. In comparison to those two studies, the model used in this study allows, on the one hand, the simulation of various traumatic injuries to the teeth and, on the other hand, it can be 3D printed within the faculty without additional commercial costs besides those for the resin.

Despite these positive results, several important limitations of this study must be acknowledged. First, the sample size of 42 students restricts the statistical power and generalizability of the findings. Second, the decline of participants in the survey after 1 year is another significant challenge as it reduces the study's statistical power, potentially limiting the ability to detect meaningful differences or effects. The voluntary nature of the survey, coupled with evolving interest among students over time, likely contributed to this reduction. Future research should therefore involve larger cohorts, ideally spanning multiple institutions, to enhance external validity. Third, this study relied on self‐reported competence and confidence measures, which may not always correlate with objective performance. Complementing self‐assessment data with objective evaluations—such as clinical performance checklists—could provide a more comprehensive understanding of student competence. As previous research shows that students' knowledge of TDI can decrease significantly within 6 months of purely lecture‐based instruction [3], mirroring similar declines observed in first aid knowledge [29], thus, future studies should include longer follow‐up assessments at even later time points to determine the durability and clinical relevance of these improvements in self‐perceived competence. In future studies, the case‐based manuscript could be replaced by a specially designed artificial intelligence chatbot as it has been described in literature [30]. This could enhance the realistic simulation and interactivity of the training.

## 5 Conclusion

This study highlights the positive and long‐lasting effects of integrating simulation‐based, hands‐on training using a realistic 3D‐printed model into the undergraduate dental curriculum to improve students' self‐perceived competence in managing TDI. The observed improvements demonstrate the potential of such educational interventions to bridge the gap between theoretical knowledge and real‐world application. Although students' concerns about treatment complications persisted, the results suggest that such hands‐on training can reinforce practical skills, increase self‐perceived competence, and potentially improve overall clinical readiness.

## Author Contributions

**Marcel Reymus:** conceptualization, methodology, investigation, formal analysis, writing – original draft, supervision. **Christina Fotiadou:** methodology, investigation, data curation, visualization, writing – review and editing. **Falk Schwendicke:** writing – review and editing. **Ralf Krug:** formal analysis, writing – review and editing. **Christian Diegritz:** conceptualization, methodology, investigation, writing – original draft.

## Conflicts of Interest

The authors declare no conflicts of interest.

## Data Availability

The data that support the findings of this study are available from the corresponding author upon reasonable request.

## Appendix 1 Case‐Based Manuscript

1. A worried mother calls your office. She reports that her 16‐year‐old son fell off his skateboard about 10 min ago. He is bleeding from the mouth and has a tooth knocked out. She asks for instructions on what to do with her son and the knocked‐out tooth.
  – What do you tell the mother?
  – What questions do you ask to obtain more detailed information about the avulsed tooth?
  – What instructions do you give for the avulsed tooth?
  – If the avulsed tooth could not be repositioned immediately, what transportation medium would you recommend?
2. Mother and son present themselves at your practice 20 min after the phone call. The knocked‐out tooth was stored in milk 5 min after the phone call.
  – What anamnestic information is important to you before starting treatment?
  – How do you proceed with the findings? What diagnostic tools do you use?
  – Which X‐ray images do you take?
  – How would you proceed if the tooth was stored in milk?

*Additional Information*

– Tooth 14: Percussion −, Sensibility +
– Tooth 13: Percussion −, Sensibility ++, Bleeding from the sulcus
– Tooth 12: Percussion −, High metallic sound by percussion, Sensibility −
– Tooth 11: Percussion +−, Sensibility ++
– Tooth 21: Empty tooth socket with blood clot within
– Tooth 22: Percussion +, Sensibility −, loosening of the tooth
– Tooth 23: Percussion −, Sensibility +
– Tooth 24: Percussion −, Sensibility +

No mobility of the alveolar process is detectable.

Region 12 dolent elevation of the mucosa is diagnosable.

Tooth fragment tooth 11 stored in milk.

3 Make a tooth‐related diagnosis and describe the therapy you would perform.
4 Perform therapy on the model.
5 At the end of the treatment, the mother asks what she and her son should pay attention to after the treatment and whether her son should take any medication.

Inform the mother about the behavior after the treatment and possible intake of medicine. Explain the necessary recall regime for each individual tooth, explain potential complications, and discuss the (long term) prognosis of each individual tooth.

6 Would your treatment have been different if the avulsed tooth 21 had been kept dry for more than 60 min? What complications might have resulted?
